# Imaging for the Initial Staging and Post-Treatment Surveillance of Penile Squamous Cell Carcinoma

**DOI:** 10.3390/diagnostics12010170

**Published:** 2022-01-12

**Authors:** Samuel J. Galgano, John C. Norton, Kristin K. Porter, Janelle T. West, Soroush Rais-Bahrami

**Affiliations:** 1 Department of Radiology, University of Alabama at Birmingham, Birmingham, AL 35294, USA; samuelgalgano@uabmc.edu (S.J.G.); kkporter@uabmc.edu (K.K.P.); janellewest@uabmc.edu (J.T.W.); 2 O’Neal Comprehensive Cancer Center, University of Alabama at Birmingham, Birmingham, AL 35233, USA; 3 Department of Urology, University of Alabama at Birmingham, Birmingham, AL 35233, USA; jcnorton@uabmc.edu

**Keywords:** penile cancer, squamous cell carcinoma, lymphadenopathy, cancer imaging, computed tomography, positron emission tomography, magnetic resonance imaging

## Abstract

Although relatively rare in the United States, penile squamous cell carcinoma is encountered worldwide at a higher rate. Initial diagnosis is often made on clinical exam, as almost all of these lesions are externally visible and amenable to biopsy. In distinction to other types of malignancies, penile cancer relies heavily on clinical nodal staging of the inguinal lymph node chains. As with all cancers, imaging plays a role in the initial staging, restaging, and surveillance of these patients. The aim of this manuscript is to highlight the applications, advantages, and limitations of different imaging modalities in the evaluation of penile cancer, including ultrasound, computed tomography, magnetic resonance imaging, and positron emission tomography.

## 1. Introduction

Squamous cell carcinoma (SCC) represents the vast majority (≥95%) of penile cancers [1]. While penile cancer is relatively rare in the United States, accounting for less than 1% of malignancies in American men, penile SCC accounts for up to 10% of all malignant neoplasms among men in Asia, Africa, and South America [2]. Penile SCC has a multifactorial etiology; an important risk factor is human papilloma virus (HPV) and high-risk subtypes, particularly subtypes 6, 16, and 18, have been found in up to 45 to 80% of cases [3,4,5,6]. Additional risk factors include smoking/tobacco use, HIV/AIDS, increased number of sexual partners, chronic inflammation of penile skin, phimosis, obesity, socioeconomic status, and psoriasis treatments (psoralen plus UVA phototherapy). Circumcision in childhood provides a protective effect, related at least in part to a reduction in phimosis [7,8].

Penile SCC can occur at any age but is most commonly seen in men between 50–70 years of age [9]. Patients usually present due to the presence of a palpable penile lesion, which may be associated with pain, discharge, bleeding, or foul odor. These lesions can be nodular, ulcerating, or fungating in appearance. Some of these lesions arising on the glans penis or inner preputial skin, may be obscured on physical exam by the presence of phimosis [5,10]. Penile SCC, from most to least common, presents on the glans (34.5%), prepuce (13.5%), and penile shaft skin (5.3%) [11]. There are four subtypes of SCC: verrucous, papillary squamous, warty, and basaloid, with the verrucous subtype conferring the lowest metastatic potential [12]. Penile SCC is staged according to clinical exam and biopsy utilizing the American Joint Cancer Committee 8th edition TNM classification (Table 1), which drives management and follow-up of these patients.

Upwards of 30% of men will present with palpable inguinal lymph nodes, of which about 60–80% represent regional metastatic spread of penile SCC, with the remainder of patients with inguinal lymphadenopathy representing an inflammatory reaction etiology [13]. Most of these penile cancers are superficial and low stage upon presentation with less than 10% of men having distant metastatic involvement upon presentation. Penile SCC spreads in a stepwise fashion, most commonly to the inguinal lymph nodes, first to superficial inguinal lymph nodes, then to deep inguinal nodes, later to pelvic nodes, and then ultimately to more distant lymphatic spread and visceral organ sites of metastases. The most common sites for distant metastasis include the lungs, liver, bone, and brain.

In this review, we present an update and overview on imaging modalities used for the primary staging, restaging, and post treatment surveillance of penile SCC. We investigate the utilization of different imaging modalities such as ultrasound (US), computed tomography (CT), magnetic resonance (MRI), and positron emission tomography (PET) imaging.

## 2. Ultrasound

### 2.1. Primary Staging

Ultrasound may be the initial imaging modality used if a penile mass is identified, largely due to widespread availability, low-cost, and ability to perform in nearly every healthcare setting. Owing to the superficial nature of the penis, ultrasound can readily be used to evaluate abnormalities of the penis, most commonly in cases of suspected fracture and Peyronie’s disease [14,15]. Penile masses are less commonly encountered than other entities, but the potential role of ultrasound has been proposed for decades [16,17]. Due to the high spatial resolution, ultrasound is capable of clearly delineating the local extent of tumor within the penis and establish potential invasion into the corpora and/or urethra. This information may be useful in establishing a surgical plan, particularly if there is consideration between a partial and total penectomy.

However, a more widespread utilization of ultrasound is in evaluation of inguinal lymphadenopathy. In patients with palpable or suspected inguinal adenopathy, ultrasound is a first-line imaging tool utilized to both diagnose and guide potential percutaneous biopsy. In cancers that commonly metastasize to inguinal lymph nodes, including vulvar cancer, there has been considerable work on standardizing descriptors to more accurately report level of suspicion [18]. In patients with penile cancer, the presence of inguinal lymph node metastases indicates the need for either a unilateral or bilateral inguinal lymph node dissection. Thus, a non-invasive method of establishing inguinal lymph node metastases in these patients is of high clinical importance. However, ultrasound is insensitive for inguinal lymph node metastases unless lymph nodes are pathologically enlarged and a negative inguinal ultrasound does not obviate the need for lymph node dissection [10,19]. However, in patients with pathologically enlarged lymph nodes, ultrasound-guided core biopsy or fine-needle aspiration may be useful in confirming a diagnosis prior to proceeding to surgery as part of establishing the surgical plan.

### 2.2. Restaging and Post-Treatment Surveillance

Following surgical resection and inguinal lymph node dissection, the National Comprehensive Cancer Network (NCCN) and European Association of Urology (EAU) guidelines recommend serial clinical exams to evaluate for recurrence [20]. Unlike other malignancies such as thyroid cancer and melanoma, surveillance ultrasound of the postsurgical nodal basins is not recommended in penile cancer. However, if a local recurrence is suspected in the penectomy bed or inguinal region, NCCN guidelines recommend a percutaneous lymph node biopsy to confirm a diagnosis in patients who have not undergone prior inguinal lymphadenectomy or radiation therapy [20]. Given the superficial nature of these lymph nodes, ultrasound-guided biopsy is frequently the modality of choice in these patients. If a lesion in the penectomy bed is palpable, but not easily visualized on the skin surface, ultrasound-guided biopsy may also be utilized to confirm local recurrence.

## 3. Computed Tomography

### 3.1. Primary Staging

Computed tomography (CT) of the chest, abdomen, and pelvis is the most commonly available and most frequently utilized imaging modality for patients with cancer. Although not able to be done portably like ultrasound, CT provides high-contrast images of the soft tissues of the body with high spatial resolution and makes an excellent imaging modality for the detection of metastatic disease. CT is a widely available imaging modality and relatively low-cost in comparison to advanced imaging modalities such as magnetic resonance imaging (MRI) and positron emission tomography (PET). CT utilizes ionizing radiation to obtain images, but the amount of radiation received from medical imaging in patients with cancer is unlikely to cause harm in the vast majority of patients [21,22].

**Figure 1:**
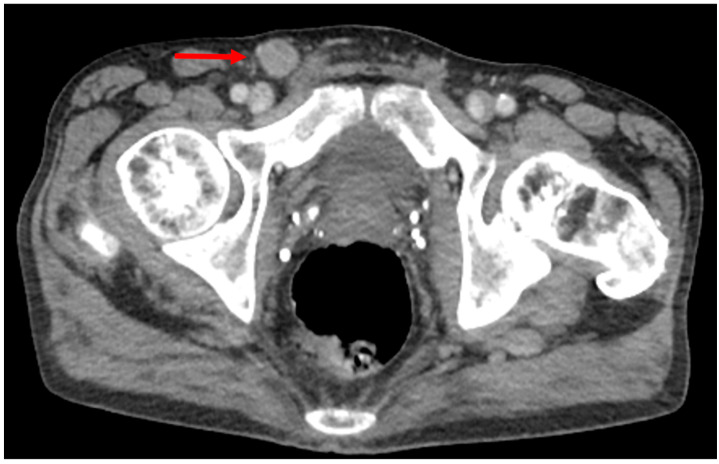
55-year-old male with newly diagnosed penile cancer presents for initial staging CT of the abdomen and pelvis. Axial contrast enhanced CT through the level of the pelvis demonstrates an abnormally rounded, enlarged right inguinal lymph node (arrow) that was confirmed to be metastatic at time of surgery.

Initial management strategies and imaging workup in patients with penile cancer is largely driven by the tumor stage and clinical nodal status [10]. Per NCCN guidelines, not all patients with newly diagnosed penile cancer should undergo staging with cross-sectional imaging [20]. For patients with early-stage tumors up to T1a and nonpalpable inguinal lymph nodes, NCCN guidelines do not recommend imaging of the chest, abdomen, and pelvis with CT. However, for patients with tumors classified as T1b or greater or patients with palpable inguinal lymph nodes, cross-sectional imaging of the chest, abdomen, and pelvis (typically CT) is recommended for systemic staging and evaluation of potential metastatic disease and iliac adenopathy (Figure 1) [10]. Studies have demonstrated value in preoperative CT in patients with high-risk pathological node positive penile cancer, with the presence of central nodal necrosis and/or an irregular nodal border seen more commonly among high-risk patients [23]. However, as with ultrasound, a known limitation of CT is the inability to accurately detect metastatic disease in lymph nodes that are not pathologically enlarged. Thus, as inguinal lymph node metastases are the most common site of metastatic disease in patients with penile cancer, CT is typically only able to detect lymph nodes that are pathologically enlarged and suffers from similar limitations to ultrasound. However, while ultrasound is only able to provide a targeted evaluation of an area of concern, CT allows for quick, comprehensive whole-body staging in patients with many types of cancer.

### 3.2. Restaging and Post-Treatment Surveillance

Following definitive treatment, surveillance CT of the chest, abdomen, and pelvis is recommended for patients with N2 or N3 disease [20]. In these patients, chest CT is recommended every 6 months for a total of two years while abdominopelvic CT is recommended every three months for the first year, then transitioning to every six months thereafter for a total of two years. In patients who have not undergone inguinal lymph node dissection or radiotherapy, a goal of surveillance imaging would be to detect disease early in the inguinal region while the patient may still benefit from local therapies, including lymphadenectomy, chemotherapy, radiation therapy, or a combination thereof. Alternatively, for those patients who develop distant metastatic disease, CT becomes a cornerstone of their oncologic imaging algorithm in monitoring response to systemic chemotherapy.

## 4. Magnetic Resonance Imaging

### 4.1. Primary Staging

The superior soft-tissue contrast, multiplanar capabilities, and excellent spatial resolution of MRI make it the imaging modality of choice for local staging of penile cancer. Although US employing high-frequency transducers has better soft-tissue resolution when compared to CT, MRI is superior for local staging. MRI may be used to evaluate the tumor depth of invasion, local extent of tumor (particularly when it involves the base of the penis), and involvement of the tunica albuginea and other adjacent structures. While staging has been traditionally through clinical examination, MRI is superior to physical examination for local staging, particularly for assessing involvement of the corpora cavernosa [24]. If depth of tumor invasion cannot be determined by physical examination or if a patient presents with palpable lymphadenopathy, MRI may be used to accurately evaluate the extent of disease, improve surgical planning, and facilitate election of more conservative penile preserving surgical treatments [25]. Additionally, MRI may also be used for postoperative surveillance for patients who have undergone partial penectomy.

**Figure 2:**
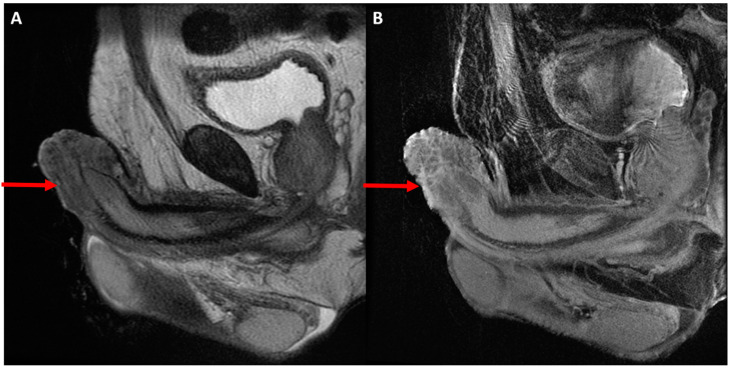
51-year-old male with newly diagnosed penile cancer of the glans penis presents for MRI to evaluate the depth and degree of local invasion and for surgical planning. Sagittal T2-weighted (**A**) and T1 fat suppressed postcontrast (**B**) images demonstrate the known mass at the glans penis with intermediate T2 signal intensity and hypoenhancement invading into the adjacent corpora. Of note, the degree of corporal invasion is more clearly demonstrated on postcontrast images in this case.

On MR imaging, penile SCC are typically infiltrating masses that are hypointense relative to the corpora on both T1-weighted (T1WI) and T2-weighted images (T2WI) and hyperintense relative to fascia and the tunica albuginea on T2WI. Small field of view (FOV) T2WI are most important for local T-staging and assessment of adjacent structure involvement, including the corpus spongiosum (Category T2 by American Joint Committee on Cancer (AJCC) 8th edition TNM staging, 2017), corpus cavernosum (T3), and scrotal skin, pubic bone, or prostate (T4) (Figure 2) [3,24,26]. MRI can also evaluate urethral involvement, which while no longer relevant to local staging, may alter surgical management techniques and impact patient morbidity [3].

Larger FOV MRI of the pelvis completes staging by assessing for nodal disease in inguinal and pelvic lymph nodes, which is the most significant prognostic factor in penile SCC [3]. Beyond assessing for nodal disease involvement, MRI helps inform the decision regarding lymph node dissection by accurately staging the primary cancer. For example, surveillance may be opted in lieu of lymph node dissection for patients with Tis, Ta or T1a disease, while a modified or radical inguinal lymphadenectomy may be performed for intermediate (T1b, G1-2) or high risk (T1b, G3-4; T2 or greater) disease [20].

### 4.2. Restaging and Post-Treatment Surveillance

Over 90% of all penile SCC recurrences occur within five years and greater than 70% occur within the first two years; therefore, short interval, frequent initial follow up with physical examination of the penis and inguinal region is recommended [3,20]. MRI is useful for evaluation of local recurrence, particularly if the patient is obese or if initial treatment was organ sparing. MRI can assess whether the recurrence involves the corpora cavernosa, which would necessitate a partial or total penectomy.

## 5. Positron Emission Tomography

### 5.1. Primary Staging

Given the prevalence of lymph node metastases and suboptimal detection of nodal metastatic disease with other cross-sectional imaging modalities, there is considerable interest in the use of positron emission tomography (PET)/CT for staging in patients with penile cancer. The most common radiotracer used in PET/CT for oncologic imaging is [18F]fluorodeoxyglucose (FDG), which functions as a glucose analog and demonstrates increased uptake in cancerous lesions secondary to upregulation of glucose metabolism. Following initial injection of radiotracer, there is an uptake phase of approximately one hour to allow for appropriate biodistribution of radiotracer. This tracer is excreted in the genitourinary tract, an important aspect to note given that high concentrations of excreted radiotracer in the bladder and external contamination of radioactive urine on the penis and skin surface can mimic or even obscure malignancy. Additionally, reactive inguinal lymph nodes are commonly seen due to inflammation and can mimic malignancy on FDG-PET/CT. Currently, the NCCN guidelines state that PET/CT can be used interchangeably with CT and MRI and may be the imaging modality of choice in patients with impaired renal function or allergies that prohibit the administration of intravenous contrast.

An initial study evaluating the use of FDG-PET/CT for staging patients with both recurrent and newly diagnosed penile cancer enrolled 13 patients and found a sensitivity and specificity of 75% for detection of the primary lesion with better performance for the detection of lymph node metastases [27]. Additional studies have had similar results with high sensitivity, specificity, positive predictive value, and negative predictive value for use of PET/CT in evaluation of inguinal lymph node metastases [28]. However, subsequent studies have not demonstrated the same degree of performance. Several separate prospective studies evaluating the use of FDG-PET/CT in patients with newly diagnosed penile cancer found low sensitivity of PET/CT for detection of micrometastatic disease, indicating the need for ongoing surgical staging [29,30]. A meta-analysis from 2012 found that due to low sensitivity (particularly in clinically node-negative patients), routine use of FDG-PET/CT is not justified, but that patients with clinically positive lymph nodes may benefit from PET/CT [31]. However, a Danish study prospectively compared FDG-PET/CT to contrast-enhanced CT in 171 patients and found a significant improvement in diagnostic accuracy of FDG-PET/CT compared to CT alone [32]. The same group also explored the use of FDG-PET/CT in combination with sentinel node biopsy localized by lymphoscintigraphy, which led to only a 5.6% false negative rate of sentinel node biopsy in patients who were clinically node negative [33].

There has also been some work in evaluating the use of FDG-PET/CT as a prognostication tool in patients with penile cancer. A study evaluating the use of FDG-PET/CT for prognostication found that patients with higher maximum standardized uptake values in the primary lesion and lymph node metastases were correlated with cancer aggressiveness and cancer-free survival [34]. A separate study enrolling patients with clinically node positive penile cancer found that patients with clinical N2 and N3 disease and positive FDG-PET/CT scans had overall mortality and that PET/CT could be used to stratify clinically node positive patients and those who may benefit most from neoadjuvant chemotherapy prior to surgical resection [35]. Thus, while PET/CT appears to offer potential advantages in staging and prognostication of patients with newly diagnosed penile cancer, it appears unable to replace surgical staging at this time.

### 5.2. Restaging and Post-Treatment Surveillance

**Figure 3:**
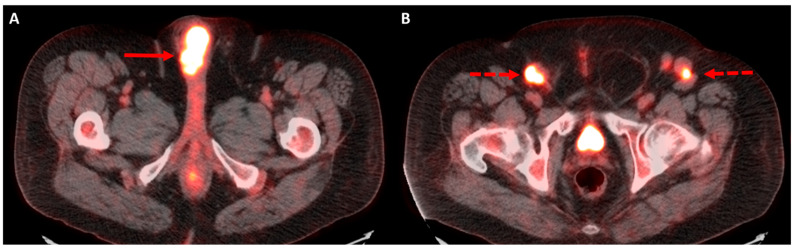
66-year-old male with history of penile cancer status post penectomy presents with suspected recurrence on clinical exam. Restaging FDG-PET/CT demonstrates an intensely hypermetabolic mass at the penectomy bed (**A**, solid arrow) and bilateral hypermetabolic inguinal lymph node metastases (**B**, dashed arrows).

Similar to CT, PET/CT is recommended by the NCCN guidelines as an option in patients with recurrent or metastatic penile cancer requiring systemic staging (Figure 3) [20]. As stated previously, this option may be best for patients with diminished renal function or allergies that prohibit the administration of intravenous iodinated contrast. However, PET/CT is typically more expensive of an imaging test than others and availability may be limited in relation to CT. A separate limitation of PET/CT for systemic staging is that these exams are often performed without intravenous contrast, which makes size measurements of visceral metastases challenging. However, in cases where new and/or indeterminate lesions exist, PET/CT can be a useful problem-solving tool to evaluating if a new finding is due to malignancy or other etiologies. As stated previously, urinary excretion of FDG and external contamination may limit detection of locally recurrent disease at the penectomy site.

## 6. Conclusions

Penile cancer is somewhat of a unique cancer in that the treatment and imaging algorithm heavily relies on clinical examination, particularly of inguinal lymph nodes. Although a relatively rare cancer, it is important for radiologists to understand what drives imaging of these patients and options that exist for staging and restaging these patients. Importantly, while inguinal lymphadenectomy remains standard-of-care for surgical staging, it carries with it an operative risk of morbidity and patients may elect to not undergo the procedure. In these patients, particular attention should be made on follow-up examinations to the inguinal nodal chains to evaluate for metastatic disease. Finally, novel combinations of molecular imaging and sentinel node biopsy offer the potential for disease prognostication and improve the ability to stratify which patient populations benefit from certain treatment approaches more than others.

## Figures and Tables

**Table 1:** American Joint Committee on Cancer 8th Edition TNM Pathologic Staging of Penile Squamous Cell Carcinoma.

Stage	Description
*Tumor (T)*	
Tx	Cannot be assessed
T0	No evidence of primary tumor
Tis	Carcinoma in situ
Ta	Noninvasive localized SCC
T1	Invasion of subepithelial connective tissue (varies by location)
T2	Invasion of corpus spongiosum with or without urethral invasion
T3	Invasion of corpora cavernosum with or without urethral invasion
T4	Invasion of other adjacent structures (scrotum, prostate, bone)
*Lymph node (N)*	
Nx	Cannot be assessed
N0	No regional lymph node metastasis
N1	≤2 unilateral inguinal nodal metastases, no extranodal extension
N2	≥3 unilateral or bilateral inguinal nodal metastases
N3	Extranodal extension of any lymph node metastasis
*Metastasis (M)*	
Mx	Cannot be assessed
M0	No evidence of distant metastasis
M1	Distant metastasis

## Data Availability

Not applicable.
